# On the generalizability of same-day partial knee replacement surgery—A non-selective interventional study evaluating efficacy, patient satisfaction, and safety in a public hospital setting

**DOI:** 10.1371/journal.pone.0260816

**Published:** 2021-12-07

**Authors:** Magnus Tveit

**Affiliations:** Department of Orthopedics, Skåne University Hospital, Clinical Sciences, Lund University, Lund, Sweden; Prince Sattam Bin Abdulaziz University, College of Applied Medical Sciences, SAUDI ARABIA

## Abstract

**Purpose:**

Programs referred to as Fast-Track/Rapid Recovery/Enhanced Recovery After Surgery have proven both effective and safe in joint replacement surgery, to the degree where same-day discharge (SDD) has been attempted in carefully selected cases at specialized outpatient units. Therefore, the primary aim of this study was to evaluate a same-day surgery protocol regarding safety using the minor partial knee replacement (PKR) procedure by non-selectively recruiting patients at a public hospital for one consecutive year.

**Methods:**

33 unselected PKR cases were included in this open clinical trial. The inclusion/exclusion criteria were solely based on logistics, as all the procedures were medial PKRs, designated the first morning slots, and performed by one single-surgeon. Strict postoperative criteria based on vital parameters, urinary function, bleeding, and mobilization had to be met before discharge was considered. SDD rate, patient satisfaction, number of outpatient visits, adverse events and readmissions within 90 days were evaluated. A predetermined subgroup analysis was also conducted where patients <80 yrs. and with an American Society of Anesthesiologists (ASA) classification <III was compared with those aged ≥80 yrs. and/or ASA class ≥III.

**Results:**

29 of 33 (88%) successfully achieved SDD. In a univariate comparison, 100% of the patients <80 yrs. and ASA class <III achieved SDD, whereas a corresponding 43% applied for those aged ≥80 yrs. and/or ASA class ≥III (p = 0.001). A 93% overall satisfaction rate was reached. Only 8% extra outpatient visits were required, all occurring within the first 2 weeks (well in line with routine practice.) One plausible transient ischemic attack and one readmission caused by a penetrating trauma not affecting the knee were identified, both of which happened 10 weeks after surgery. No adverse events or readmissions occurred within the first 48 hours of surgery.

**Conclusion:**

When following strict criteria for discharge, same-day partial knee replacement surgery may be both feasible and safe, even without preselection of patients.

## Introduction

What started as an attempt to target all aspects of perioperative care to more effectively reduce costs and complications [1], has since evolved to programs nowadays referred to as Fast-Track, Rapid Recovery or Enhanced Recovery after Surgery (ERAS). The orthopedic field was one of the early adopters and, as consequence, has since both seen a dramatic drop in length of stay (LOS) and an increased patient satisfaction after surgery [2]. Even so-called rapid discharge protocols (RDP) allowing discharge on the day of surgery (DOS), also referred to as same-day discharge (SDD), have been implemented in carefully selected joint replacement surgery (JRS) cases at specialized outpatient units [3]. Although SDD procedures in general are considered safe, one large registry-based study found both age >80 yrs. and American Society of Anesthesiologists (ASA) classification ≥III constitute a higher risk of 30-day complications and/or readmissions, both in total hip replacement (THR) and total knee replacement (TKR) surgery, whereas only a trend was found in the much less frequently performed partial knee replacement (PKR) surgery [4]. Another systematic review comparing in- and outpatient surgery regarding complications evaluated 805 original studies, of which only 17 passed the inclusion criteria, none was a randomized controlled trial (RCT), and only four were controlled [5]. Old age and “comorbidity” were found to be associated with early complications, but with indications of no between-group differences in clinical outcomes [5]. To further recognize that SDD may not be realistic for everyone, US-based JRS data have shown the same-day surgery proportion to be less than ten percent [4, 6, 7].

Knee replacement surgery has proven successful in reducing pain and improving function for those suffering from severe degeneration of the knee [8]. TKR and PKR are both suitable options for late-stage isolated medial knee osteoarthritis and osteonecrosis of the knee [9, 10]. With the correct indication and optimized volume/usage [11], the PKR procedure has not only been associated with lower postoperative LOS, but fewer adverse events and lower overall costs than the TKR procedure [2, 12–14]. Yet, unadjusted national joint registry data are consistent in showing higher revision rates for the PKR procedure [15–19]. Such contradictions probably contribute to the variation seen in PKR usage between countries, illustrated by an around 5% PKR incidence rate in both the USA [18] and Australia [19], 10% in the UK [16] and Sweden [15], and 20% in Denmark [17]. Regardless the apparent divergence between countries, the global numbers are substantial.

To further optimize the perioperative JRS pathway, with potential gains in both satisfaction [2] and costs [2, 13, 14], the minor PKR procedure seems the natural candidate when aiming to investigate whether SDD may be more easily repeatable. One deviation from current practice, which traditionally includes prompt commencement of knee flexion, was highlighted in two recently published articles on PKR procedures where delayed flexion [20] or even absence of supervised physiotherapy (other than to facilitate safe walking with crutches) [21] were evaluated. Neither study found any negative effect on range of motion, even in the short-term perspective.

There is no strong evidence in the literature suggesting that same-day surgery is generalizable, neither in terms of which patients it is suitable for nor in which hospital settings it can be safely applied. Thus, the primary aim of this study was to prospectively evaluate a same-day surgery protocol for PKR regarding safety by unselectively recruiting patients at a public hospital. The study was designed using strict postoperative criteria for discharge, where the non-selective approach that was used presumed the protocol to be firmly tested. Secondary aims were to evaluate patient satisfaction and feasibility of SDD. With few exceptions, studies on SDD have been selective, using varying cut-off levels for age and/or “comorbidity” as the common denominator. For descriptive comparison only, as the study was only marginally powered to detect any subgroup differences, a predetermined two-arm analysis with one arm based on similar selection criteria as in the literature was also intended (in the current study set to age <80 yrs. and ASA class <III).

The author hypothesized the protocol to be (i) safe (i.e., resulting in no adverse events associated with the SDD routine), (ii) patient satisfactory, and, although designed as non-selective, also (iii) to be feasible (i.e., resulting in a high overall SDD success rate combined with a low overall extra outpatient visit rate).

## Materials and methods

Primarily descriptive, this 3-month intervention designed study was conducted at Trelleborg Hospital in Region Skåne, the southernmost county council in Sweden. Around 1,500 JRSs are performed each year at the facility. Eligibility to be considered for a medial PKR was either antero-medial osteoarthritis or spontaneous osteonecrosis of the knee [9, 10].

**Fig 1:**
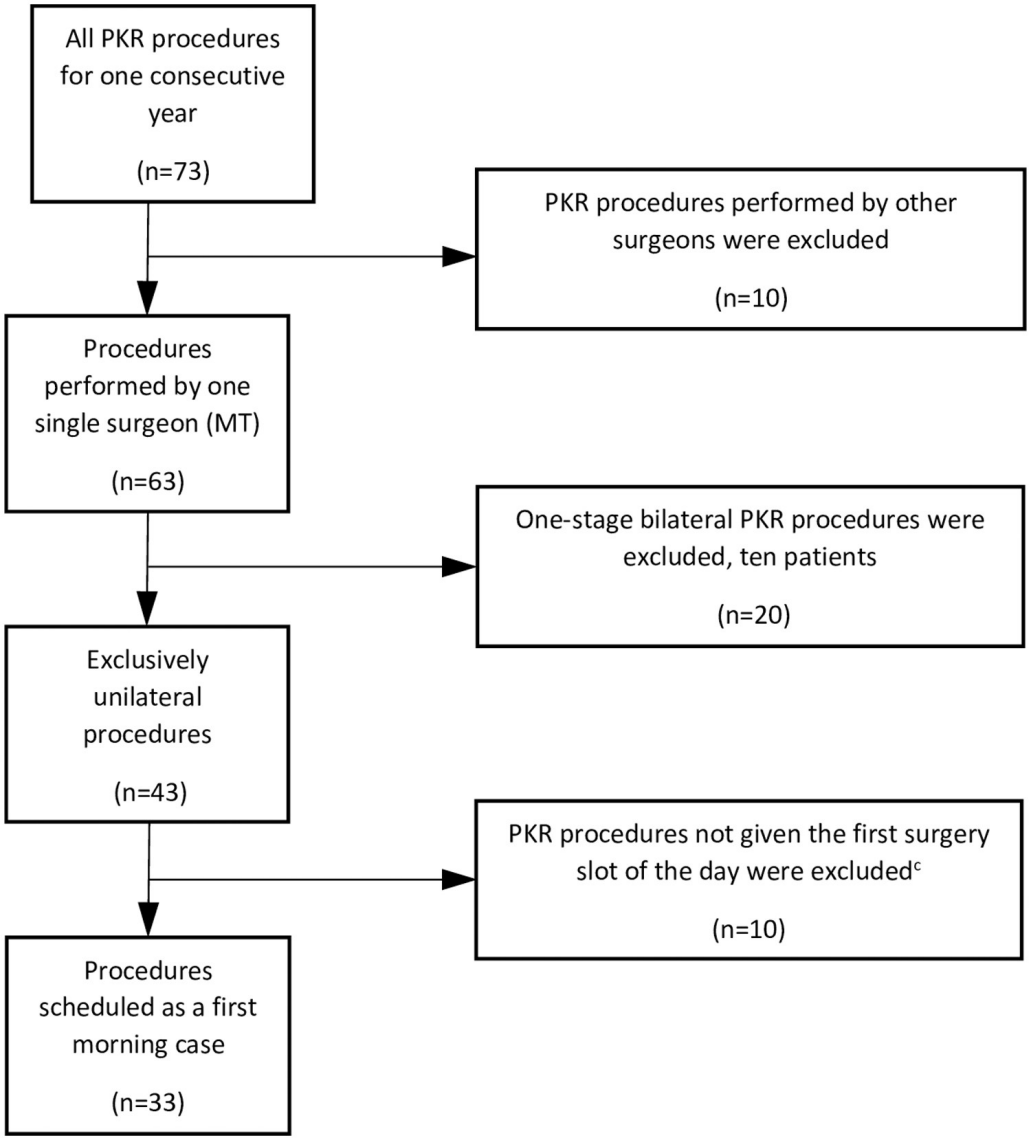
Flowchart of all the PKR procedures.^a^ performed for one consecutive year at Trelleborg hospital^b^, and the path for inclusion in this interventional study. ^a^They were all diagnosed either as antero-medial osteoarthritis or osteonecrosis of the knee, no lateral procedures were performed during this timeframe. ^b^A public hospital within Region Skåne, the southernmost county council of Sweden. ^c^The first morning slots were filled continuously as the patients were scheduled for surgery. When all slots had been taken within one week, any additional PKR cases were assigned to surgery later in the day the same week. The operation scheduling was put together on a weekly basis, in a strict chronological order, by a team of nurses and with no insight of the surgeon in question. (Prior to surgery, no preselection was made as regarded patient characteristics–everyone was considered eligible for inclusion).

The study protocol had a pragmatic approach to which of the PKR candidates would be considered for same-day surgery, as no preselection of patients was done prior to surgery. The only patient-related exclusion criteria were whether the patient was neither clinically nor radiographically qualified for a PKR procedure [9, 10]; everyone else was informed prior to surgery by a multidisciplinary team (anesthesiologist, nurse, physiotherapist, and surgeon) that if the surgery was to be scheduled in the morning, he/she would then be following a same-day surgery protocol and likely be discharged to home later the same day. The inclusion/exclusion criteria for this open clinical trial were hence based strictly on logistics as all surgeries were (i) exclusively medial unilateral, (ii) designated the first morning slot, and (iii) performed by one high-volume surgeon (MT) (Fig 1). For one consecutive year, the morning slots were filled continuously as the patients were registered for surgery. When all slots had been taken within one week any additional PKR cases were assigned to surgery later in the day the same week (as would all THRs and TKRs during this period), and consequently not included in this study. The operation scheduling was put together on a weekly basis, in a strict chronological order, by a team of nurses without any insight from the surgeon in question. Of the 73 PKR cases performed in our department within the timeframe of this study, the algorithm (Fig 1) rendered an inclusion of 33 non-selected cases. The patients were recruited from Feb 7, 2019 to Feb 14, 2020, with the follow-up of the last patient on May 18, 2020. The included patients had a mean age of 66 yrs., a mean body mass index of 28, 18% were classified as ASA class ≥III and 52% were females (Table 1).

**Table 1:** Patient characteristics and type of degenerative knee disease^a^.

	*n* = 33
	(mean ± SD or n (%))
Age (years)	65.6 ± 8.3
Female (%)	17 (51.5)
Height (cm)	173.1 ± 11.5
Weight (kg)	84.2 ± 15.8
Body mass index (kg/m^2^)	28.0 ± 3.2
ASA class ≥III (%)	5 (15.2)
Charnley class C (%)	9 (27.3)
Antero-medial osteoarthritis (%)	27 (81.8)
Osteonecrosis of the knee (%)	6 (18.2)
Other knee diseases (%)	0 (0.0)

^a^ Descriptive data are presented as unadjusted means with standard deviations (SD) or as proportions (%).

**Fig 2:**
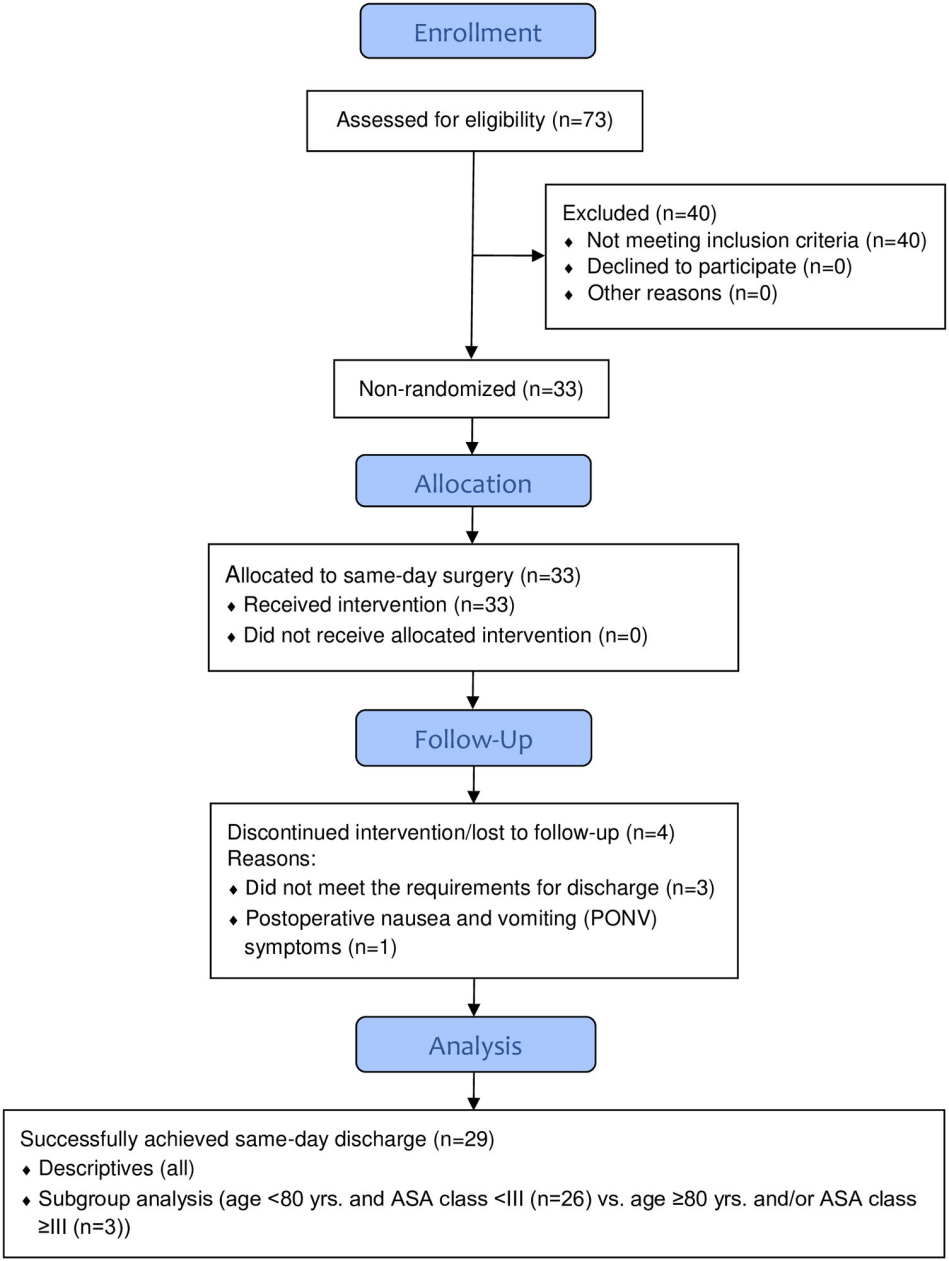
TREND flow diagram.

The patients were also separated into two predetermined subgroups based on age and ASA class: those aged <80 yrs. and ASA <III and those aged ≥80 yrs. and/or ASA ≥III (Fig 2). The 80-year cut-off might be perceived as aggressive but was chosen as it has been reported to be a significance level for short-term complication/readmission in the literature [4]. (The 80-year cut-off also happened to correspond with the 90^th^ percentile of the patients who had received a PKR within Region Skåne in the last five years.) The subgroup analysis was a deviation from the original study protocol (S1 Appendix).

**Fig 3:**
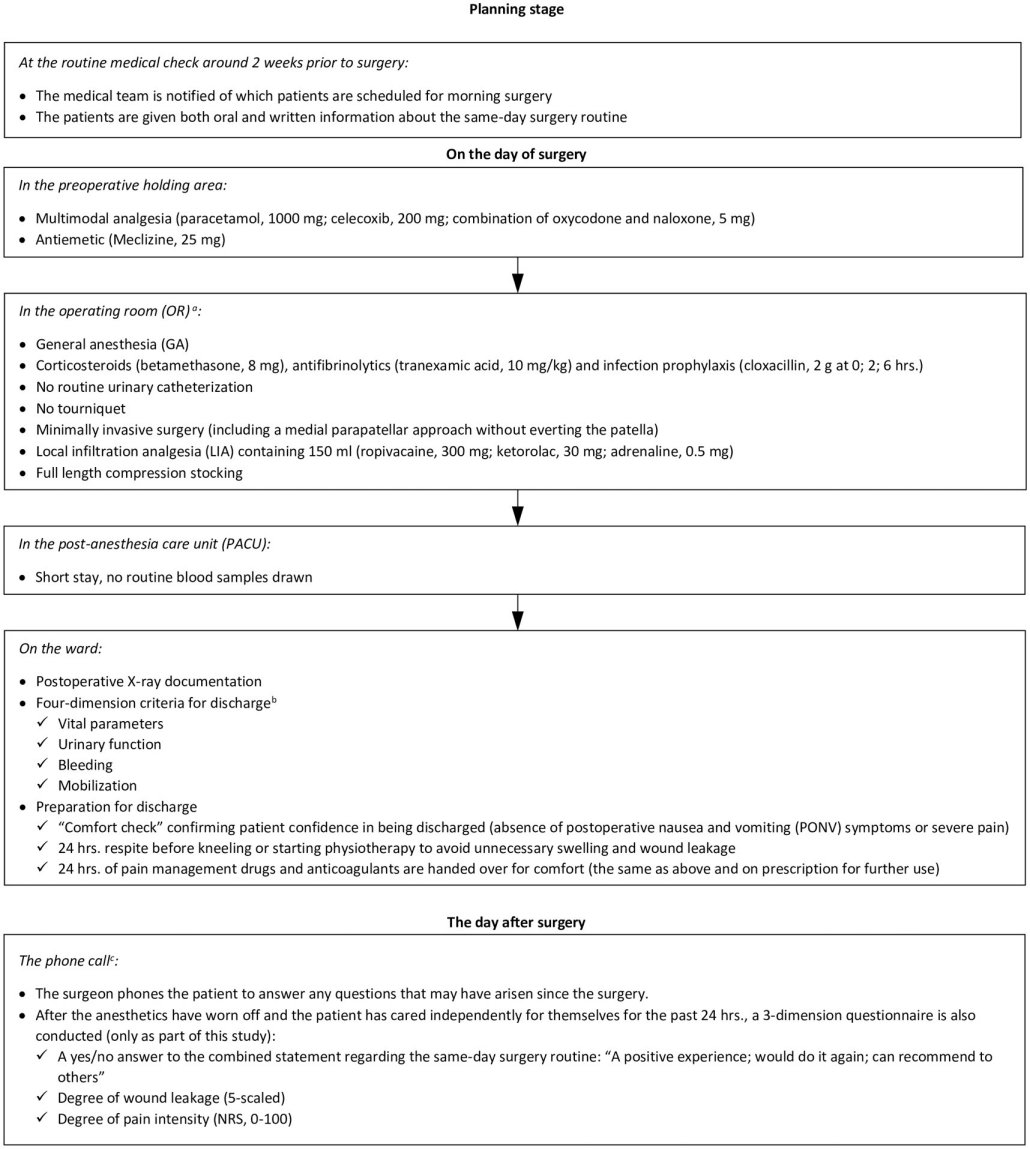
The algorithm for the same-day surgery protocol. ^a^The perioperative regime aimed at optimizing for rapid mobilization and avoiding early postoperative numbness (GA), urinary retention (no catheter), bleeding (antifibrinolytics and compression stockings), stress (corticosteroids), and discomfort (LIA and logistics). ^b^As no preselection of patients was made, it was all the more important to have strict criteria for discharge: Vital parameters were measured and scored by the National Early Warning Score (NEWS), where the threshold was set to NEWS 0. As for the urinary function, less than 200 ml of residual volume after spontaneous void was required. No wound leakage was allowed, and a physiotherapist made sure that the patient could perform ADL safely before discharge could be considered. ^c^It was anticipated that not all information would be comprehended right after surgery and questions might arise, which is why the protocol emphasizes that the surgeon must always call the day after surgery to answer any questions and to evaluate any early signs of complications.

All surgeries were performed with minimally invasive technique using the LINK^®^ Sled prosthesis. Other key elements of the surgery routine involved the use of general anesthesia and administration of local infiltration analgesia (LIA), however, neither urinary catheter nor tourniquet was used (Fig 3).

With solely logistical modifications to an existing ERAS protocol, a same-day surgery protocol was designed (Fig 3). Using this protocol, the patients were discharged on DOS if all the postoperative criteria for discharge had been met, and the patient felt confident to do so (as opposed to one or more days after surgery for the routine ERAS protocol). It required a synchronized effort by the multidisciplinary medical team to have every criterion checked and at the same time not stress the patient. Additionally, prior to surgery the patients were repeatedly informed about the SDD routine as all members of the team gave their perspectives. Such clear patient expectations were reported as crucial for successful SDD achievements [22, 23]. The postoperative criteria for discharge were categorized into four dimensions–*vital parameters*, *urinary function*, *bleeding*, and *mobilization*. The vital parameters were measured and scored using the National Early Warning Score (NEWS) [24], where the total possible score ranges from 0 to 20 and the higher the score the greater the clinical risk. The threshold for discharge in this study was conservatively set to NEWS 0. The urinary function algorithm for discharge on DOS was, in short, if there was no need for catheterization and less than 200 ml of residual volume after spontaneous void. A compression stocking, which was used to reduce swelling and potentiate the effect of the LIA administrated during surgery [25], was removed six hours post-surgery. Then, if there was no ongoing bleeding, discharge could be considered. Last but not least, an inpatient physiotherapist (PT) evaluated whether basic activities of daily living (ADL) could be performed safely using crutches, i.e., without any focus on a particular range of motion (which instead would first be checked at the two-week nurse/PT outpatient appointment). The PT stayed at the hospital until 4 p.m., which was the time limit for discharge evaluation regarding mobilization, whereas the time limit for the other postoperative discharge criteria was 6 p.m. Patients who did not meet discharge criteria in all four dimensions in time were held overnight and discharged the day after surgery. With the aim of minimizing discomfort in the first critical 24 hours, all patients who met the criteria and were discharged on DOS were handed an equivalent dose of analgesics as on the prescription. To further minimize the pain and the risk of wound leakage, the patients were recommended not to start exercising the knee at all within the first 24 hours after surgery.

The surgeon phoned all the patients the day after surgery for an early medical check. As part of the secondary aims of this study, a three-part survey was then also conducted regarding patient satisfaction, wound leakage, and pain intensity (Fig 3). Pain was assessed using a validated score, the Numerical Rating Scale (NRS) [26], whereas the other two (satisfaction and wound leakage) were assessed using anchor type questions (S2 Appendix), not yet validated. Therefore, the threshold for a patient to be marked as “satisfied” was set conservatively in that a positive response was required to all three of the statements “a positive experience; would do it again; can recommend to others”, i.e., a binary yes/no evaluation. Similar anchor type questions have been used in other studies [14, 20]. The five-level ordinal type question regarding wound leakage had been thoroughly pre-tested as it was copied straight from clinical practice at the study site, where patients with bandage saturated more than 50% (level 3 and above) are recommended to have it changed. To best evaluate the frequencies of additional outpatient visits, with a plausible association to the same-day surgery routine, the same policy was strictly followed in the current study.

The 3-month follow-up ended with a doctor’s appointment confirming little to no discomfort, no need of crutches, and a clinically well-balanced knee with full range of motion (otherwise action taken). The proportion of SDD achievements and any additional resources relocated to the outpatient department, up to or initiated at either of the two-week nurse/PT appointment and/or the 3-month doctor’s appointment, constituted the feasibility measures that summarized the secondary aims of this study. Determining 30- and 90-days adverse events (AEs) [27], i.e., complications that may have had causal association with the surgery, and readmissions constituted the primary aims of this study. These were confirmed by interviewing and re-reading all the medical reports. Cohort descriptives and subgroup univariate analyses were conducted for all outcome variables. In addition, with SDD as the dependent variable, a stepwise logistic regression (Forward: Likelihood Ratio) was also conducted to retrospectively predict those who would be able/unable to achieve SDD.

### Statistics

Descriptive data are presented as unadjusted means with standard deviations (SD), medians with range, or as frequencies with percentages (%). As a measure of dispersion for the overall descriptive endpoint percentages, the Clopper-Pearson method was used. The between-group differences are presented as means with 95% confidence interval (95% CI) distributions (using Welch’s t-test for all variables). The p values were calculated using Welch’s t-test, the Mann–Whitney U-test and Fisher’s exact test for numerical, ordinal, and categorical variables respectively. A p value less than 0.05 was considered statistically significant. The multivariate analysis was conducted using logistic regression. Furthermore, the statistical analyses were based on unequal sample sizes and normal distribution, the latter visually evaluated using Q–Q probability plots. Data analyses were conducted using SPSS^®^ Statistics, Version 25 (IBM^®^, Armonk, New York, USA).

A pre-study power analysis showed that 25 patients were needed to detect a statistically significant 60% between-group incidence difference with an 80% power and an alpha-level of 0.05. The analysis was based on a 4:1 ratio assumption for the subgroup enrollment, with a success rate of 80% and 20% respectively.

### Ethics

This interventional study (open clinical trial) was carried out in compliance with the 7^th^ version (2013) of the Helsinki Declaration and in accordance with the TREND Statement (www.cdc.gov/trendstatement). The study was approved by the Swedish Ethical Review Authority (2020–02000) as of Aug 4, 2020 and by the regional Ethical Review Board of Region Skåne (172–20) as of Sep 29, 2020. Written informed consent was obtained from all participants.

One can argue the current study to be observational cohort designed, as opposed to an interventional open clinical trial; at least this was the original opinion of the author and the reason for not having registered the study before enrollment of participants. The author has accepted the arguments forwarded by PLOS ONE and, in accordance with its guidelines, the study has since been registered at ClincalTrials.gov (NCT04790591).

## Results

No patient suffered any AE or readmission within 48 hours of surgery, or even within 30 days of surgery (Table 2). At ten weeks after surgery one patient experienced an AE caused by a plausible transient ischemic attack and one other patient, also at ten weeks, was readmitted because of a penetrating trauma not affecting the knee. Neither of these AEs were believed to have had any casual association with the surgery.

**Table 2:** Descriptives and unadjusted subgroup analyses^a^ of postoperative efficacy, patient satisfaction, and safety outcome measures.

	Unselective descriptives	Selective comparisons^b^			
	all	Age <80 yrs. and ASA class <III	Age ≥80 yrs. and/or ASA class ≥III	Between-group difference	
	(n = 33)	(n = 26)	(n = 7)		
	mean ± SD, median (range) or n (%)	mean ± SD, median (range) or n (%)	mean ± SD, median (range) or n (%)	mean % (95% CI)	P value
**The day after surgery**					
Same-day discharge achievement	29 (87.9)	26 (100.0)	3 (42.9)	-57.1 (-77.6 to -36.7)	**0.001**
Patient satisfaction^c^	27 (93.1)	24 (92.3)	3 (100.0)	7.7 (-25.0 to 40.4)	1.00
Pain (NRS, 0–100)	24.8 ± 14.8	24.6 ± 15.0	26.7 ± 15.3	2.1 (-31.5 to 35.6)	0.84
Wound leakage^d^	1 (1 to 5)	1 (1 to 4)	1 (1 to 5)	0,6 (-5.0 to 6.1)	0.80
**At 3-month follow-up**					
Extra outpatient visits^e^	5 (7.9)	4 (7.1)	1 (14.3)	7.1 (-27.9 to 42.2)	0.46
Concerns at the 3-month doctor’s app.	0 (0.0)	0 (0.0)	0 (0.0)	-	-
30-day adverse events or readmissions	0 (0.0)	0 (0.0)	0 (0.0)	-	-
90-day adverse events or readmissions	2 (7.4)	2 (7.7)	0 (0.0)	-7.7 (-18.7 to 3.3)	1.00

^a^Descriptive data are presented as unadjusted means with standard deviations (SD), medians with range or as numbers with proportions (%). The between-group differences are presented as means with 95% confidence intervals (95% CI) and p values using Welch’s t-test, the Mann–Whitney U-test and Fisher’s exact test for numerical, ordinal and categorical variables respectively.

^b^All the statistical analyses, except for the same-day discharge rates, were exclusively conducted on those who had been discharged on day of surgery (i.e., 26+3 patients).

^c^To be defined as “satisfied” with the same-day surgery routine, all three of the following statements needed to be agreed on when phoned the day after surgery: “A positive experience; would do it again; can recommend to others”

^d^When phoned the day after surgery, the patients were asked to evaluate the amount of leakage by choosing either of five categories: 1 = none or a small stain; 2 = less than half the bandage; 3 = more than half the bandage, dry; 4 = more than half the bandage, wet; 5 = bandage saturated

^e^Defined as any additional visits other than the planned 2-week nurse/physiotherapist and 3-month doctor’s appointments for each patient. (All happened within 2 weeks after surgery and were caused by wound leakage that needed change of bandage.)

A 93% satisfaction rate was achieved with a 95% confidence interval of 77–99%. Two patients stated not having been comfortable with the same-day surgery routine and would not go through it again, of which one had to return the day after surgery due to wound leakage and the other had shown skepticism even before surgery (Table 2).

Beyond the 58 planned outpatient appointments (29 same-day surgeries times 2 visits) a total of 5 (8%, 95% CI 3–18%) extra visits were required. All happened within 2 weeks after surgery and were solely caused by wound leakage, with a times-two trend if aged ≥80 yrs. and/or ASA class ≥III (p = 0.46) (Table 2).

Of the 33 unselective PKR cases, 29 (88%, 95% CI 72–97%) were discharged on DOS, with the remaining four patients discharged the day after surgery (Table 2). The violated criteria for discharged on DOS in each case comprised (i) one elderly patient who did not meet the acceptable post-void residual urine volume, (ii) one patient with multiple medical conditions who experienced severe postoperative nausea and vomiting (PONV) symptoms resulting in prolonged stay in the post-anesthesia care unit (PACU), and (iii) although not experiencing any symptoms and in fact optimistic towards being discharged on DOS, two patients with prior heart conditions were not discharged on DOS as one needed inotropic agents and the other suffered from perioperative arrhythmia, and therefore stayed overnight at the PACU.

The 26 patients aged <80 yrs. and ASA class <III were discharged on DOS in 100% of the cases, whereas the corresponding figure was 43% for the 7 patients aged ≥80 yrs. and/or ASA class ≥III (p = 0.001) (Table 2). When comparing those who did and did not achieve SDD, using univariate analyses (Welch’s t-test, the Mann–Whitney U-test and Fisher’s exact test for numerical, ordinal and categorical variables respectively), only ASA class ≥III reached significance level (p = 0.01) and age was the closest non-significant other contributor of the preoperative patient characteristics (Table 1). Age and ASA class ≥III were then included as predictor variables of SDD achievement in a logistic regression analysis, which showed that the two variables combined (= one block) accounted for 51.0% of the variability in achieving SDD. This model, which significantly outperformed the null model (p = 0.006), was also able to classify 96.6% of SDD and 50.0% of unsuccessful SDD, for an overall success rate of 90.9%. (When adding each of the remaining preoperative patient characteristics into a stepwise regression, no other model further improved the overall SDD prediction).

Even though the protocol stipulated that the patients would receive general anesthesia, 5 of the included patients were given spinal anesthesia, either mistakenly (2 patients) or on the anesthesiologist’s decision due to their medical conditions (3 patients). Despite the longer stay in the PACU after being administered spinal anesthetic (and catheterization), all 5 patients managed to meet the post-surgery criteria and were discharged on DOS.

## Discussion

The most important finding of the present study was that, despite its being designed as non-selective, no AE/readmissions occurred within 30 days of surgery, confirming the same-day surgery protocol to be safe, regardless of whether or not discharged on DOS. The 93% satisfaction rate and only 8% additional outpatient visits compared to planned (well in line with routine practice) also confirmed its feasibility. One unique aspect of the current study was that patients aged <80 yrs. and ASA class <III achieved SDD in 100% of cases (thereby indirectly acknowledging the selection criteria often used in the literature).

### Risks

A transition of the minor PKR procedure into same-day surgery appears tempting, especially as the procedure is reported to have less risk of short-term complications than does TKR [14]. A recent US database study of 169,406 patients that had undergone JRS reported no difference in readmission, adjusted for comorbidity, when comparing the inpatient with the outpatient group [7]. Data on PKR exclusively is hard to find, but one large retrospective register-based study by Bovonratwet et al that compared 5,312 inpatient and 568 outpatient PKR cases between 2005 and 2015 reported no difference in 30-day readmission rate and concluded that same-day surgery can be considered safe in carefully selected patients [6], as was also reported in non-randomized controlled trials [28–32].

Nevertheless, patients may experience health problems that have been caused by or become symptomatic because of the surgery. Preoperative patient characteristics such as old age, high ASA class, high body mass index, and female sex have repeatedly been shown to predict less likelihood of discharge on DOS after JRS, including PKR [33–36]. Recent studies have highlighted old age [4, 36, 37] and cardiovascular disease [4, 37] as predictors of undesirable outcomes such as extended LOS, complication, and readmission. The same associating factors (age, ASA class, gender, and cardiovascular disease in particular) were repeated in this study of those not being discharged on DOS. Serious cardiovascular events requiring immediate attention such as pulmonary embolism, cardiac infarction, and cerebrovascular accident are of course better handled in an inpatient scenario, yet they are extremely rare [38, 39]. With no preselection of patients, the current study highlighted the importance of following strict (and conservative) criteria for discharge (Fig 3). Neither of the two identified AE/readmissions ten weeks after surgery was likely to have had any causal association with the surgery, even less so with discharge on DOS (Table 2).

Postoperative manifestation of surgical stress such as pain, fatigue, wound drainage, and PONV–all of which hinders mobilization–are known factors that influence LOS [3, 36, 40, 41]. Glucocorticoids have shown to be most powerful in reducing these inflammatory responses [42], and high-dose corticoids on a large scale have been reported with so far no safety issues [43]. However, neither corticoids [44] nor postoperative hemoglobin concentrations or opioid use [45] seem to correlate to orthostatic intolerance.

### Feasibility

It is fair to say that a general shortening of LOS started off in the US where the healthcare structure and financial environment differs substantially from Europe. For a long time PKR, as of 2018 TKR and in 2020 THR were removed from the so-called inpatient-only (IPO) list in the US, which had great impact on the number of same-day JRS performed each year at the ever-growing ambulatory surgery centers (ASCs). A systematic review, which comprised ten US-based studies on JRS [3], including four PKR studies [22, 23, 30, 46], summarized SDD to be both feasible and safe in selected cases, with a reported 90 to 100% SDD success rate.

As for recent European-based studies, the SDD achievement rates have been reported to span from 59% to 94%, presumably to a large extent depending on each study’s unique inclusion criteria: One cohort study from the United Kingdom [47] that included 72 patients reported 85% SDD. The inclusion was, to a large extent, decided postoperatively based on “dependency” in the PACU. The included patients were mean 62 yrs. and classified as mean ASA 1.9. Logistics was reported as the main reason for not achieving SDD. Another cohort study from France [48] that included 50 patients reported 94% SDD. “Severe comorbidity” was the reason for exclusion, rendering a mean age of 67 yrs. and a mean ASA class of 2.0 for the included patients. Nausea was reported as the main reason for not managing SDD and wound leakage was reported as the main reason for the 42% extra outpatient visits needed until day ten. One non-randomized controlled trial from the Netherlands compared same-day surgery to a more traditional fast-track protocol [31]. 127 patients were screened for PKR surgery. Those with “comorbidity” and aged >70 yrs. were excluded, rendering 31% eligible for outpatient surgery– 20 in each group. 90% of those who followed the outpatient pathway achieved SDD. Logistics and uncertainty on which ASA classification the patients belonged to were reported reasons for overnight stays. One other study from the Netherlands [32], case-control designed, screened 34 patients for same-day PKR surgery. Patients with “severe cardiologic, pulmonary, and/or internal diseases” were excluded, rendering 20 patients (59%) in the outpatient group, of which 85% achieved SDD. Pain was reported as the main factor for overnight stays. In a Danish cohort study of 368 consecutive patients screened for PKR surgery [40], 69% were considered eligible for outpatient surgery. Only patients with ASA class I–II were included rendering a mean age of 64 yrs. for the included patients. 59% achieved SDD. Wound leakage and nausea were reported as the main reasons for not managing SDD.

The author could only speculate as to why the current study resulted in a 100% SDD success rate of those aged <80 yrs. and ASA class <III (Table 2). Plausible explanations for the comparably high percentages found in the current study may include a total confidence in the logistics, the preoperative multidisciplinary effort to thoroughly inform each patient about the SDD procedure, and a continued optimization of the perioperative multimodal pain management–all equally important for minimizing the physical and psychological stress that are associated with PONV symptoms [3, 36, 40, 41]. Other factors may include the protocol´s advice against early knee flexion [20, 21] and a desire for operations under general anesthesia [41], which may have further reduced the risk of pain, fatigue, swelling, and wound leakage as well as increased the chance of early mobilization right after surgery.

Few studies have evaluated the generalizability of same-day PKR surgery. With no preselection of patients, a span from 22% to 84% successful SDD achievements have been reported depending on the study [20, 36, 41, 49]. In two studies, immobilization [20, 41] was reported to be the main reason for not achieving SDD, whereas two other studies reported nausea [36, 49] to be the main reason. Even non-selectively, the 88% overall SDD success rate found in the current study was high in comparison (Table 2).

Both pain management and nausea control are key factors in same-day surgery protocols to ensure high success rates in outpatient facilities. When the generalizability of such a protocol was studied in a community hospital setting with patients undergoing PKR surgery, the discharge on DOS increased significantly from 11% to 72% [49]. Another multi-center study showed a positive association between surgeon volume/usage and rate of PKR patients discharged on DOS [50], indicating that experience may drive the demands of having a multidisciplinary same-day surgery protocol in place.

### Strengths

In its pragmatic approach, this interventional study has many strengths. First, being designed as non-selective prior to surgery, the SDD protocol (strictly based on post-surgery criteria for discharge) was thereby thoroughly tested regarding safety and demonstrated to hold up well as neither of the two identified AEs/readmissions at ten weeks were considered causally associated with the same-day discharge. The study also addressed efficacy, including the risk of merely relocating resources from the inpatient to the outpatient department while allowing discharge on DOS. Finally, patient-satisfaction was also assessed. In attempts to prospectively evaluate these three key endpoints, critical for a successful same-day surgery practice, it was considered beneficial to minimize confounding factors by having one high-volume surgeon performing all the cases. Intentionally, the algorithm for the same-day surgery protocol (Fig 3) is also presented as transparently as possible for others to compare, criticize, and adjust to refine the perioperative regime further.

### Limitations

Limitations include the non-general scenario, as the study neither addressed other types of JRS nor did it evaluate how late in the day a same-day surgery may be feasible. Although predetermined to one consecutive year, allowing the study protocol to stay static, one other weakness is the low sample size. Nevertheless, even though the current study showed a 100% discharge on DOS in the more “traditional” selective subgroup, the overall non-selective cohort demonstrated similar risk factors for extended LOS as has been reported in the literature. Also, the study lacked a control group, but this would not have been practically manageable considering the low yearly numbers. Although the current study covered 90 days compared to many similar studies on SDD with only a 30-day follow-up, one can argue that another limitation was the lack of validated post-surgery questionnaires at follow-up. This was still considered far too early after surgery to have any meaningful bearing in the long term (though all included patients will be followed with EQ-5D and KOOS 1, 6 and 10 years after surgery in accordance with a routine ERAS program running in the background). In theory, even after having re-read all the medical reports, there was still a risk of missing AEs/readmissions for which the patients might have sought (or not sought) medical attention outside the boarders of the Skåne Regional Council. However, this matter was checked at the 3-month doctor’s appointment.

## Conclusions

This study clearly demonstrated that same-day PKR surgery may be both feasible as a routine practice and safe for everyone. Age and ASA classification not only demonstrated excellent SDD prediction capabilities but were also known facts prior to surgery. The low sample size implies that the statistical significance should be evaluated with caution. Yet, if repeatability were to be crucial, inclusion criteria based on these findings may be considered.

## Supporting information

S1 Appendix Study protocol. (PDF)

S2 Appendix Three-part telephone survey. (DOCX)

S3 Appendix Three-part telephone survey (Swedish). (DOCX)

S4 Appendix TREND statement checklist. (PDF)
