# Supplementary material for: On the generalizability of same-day partial knee replacement surgery—A non-selective interventional study evaluating efficacy, patient satisfaction, and safety in a public hospital setting
Source: PLoS One. 2021 Dec 7;16(12):e0260816. doi: 10.1371/journal.pone.0260816 (PMC8651131; doi:10.1371/journal.pone.0260816)
Supplement: S1 Appendix — (PDF) [file pone.0260816.s001.pdf]

STUDY PROTOCOL

Partial knee replacement as same-day surgery within an ongoing ERAS program – an observational study on efficacy, patient satisfaction and safety in *X* unselected cases during one consecutive year

Principal investigator:
Magnus Tveit, MD, PhD
Hip and Knee Reconstruction Unit
Department of Orthopaedics
Skåne University Hospital
Lund University
SE - 221 85 Lund
Sweden
Magnus.tveit@skane.se

Other in-house clinical investigators
None

Contents

Summary 3

Background and rationale 4

Objectives 6
- Primary aims 6
- Secondary aims 6

Patients and methods 7
- Study design 7
  - Cohort study – Level of evidence, 2 7
- Patients 8
  - Inclusion criteria 8
  - Exclusion criteria 8
  - Withdrawal criteria 9
- Perioperative conditions 9
  - The operating room and medical regimes 9
  - Implant design and surgical technique 9
- The same-day surgical protocol (*in short*) 10
  - Planning stage 10
  - Day of surgery 10
  - Day after surgery 10
- 3-month follow-up 11
- Patient information 11

Data analysis 12
- Statistics 12

Ethical considerations 13
- Ethics approval 13
- Conventional X-ray 13

Administrative procedures 14
- Data management 14
- Reports and publication of results 14
- Timeline 14

Study centres 15

Surgeons 16
- Conflicts of interest 16
- Disclosures 16

References 17

Summary

Fast-track/rapid recovery/Enhanced Recovery After Surgery (ERAS) programs have shown to be proven both safe and effective in joint replacement surgery (JRS), to the degree where same-day surgery have been tested in selected cases. A transition of the minor partial knee replacement (PKR) procedure, compared with the alternative and more commonly used total knee replacement (TKR) for knee osteoarthritis (OA), into a same-day regime seems reasonable. Especially as PKR is reported to have lower risk of short-term complications than does TKR.

The aim of this study is to assess efficacy, patient satisfaction and safety outcome measures for PKR when using a same-day surgery protocol in a Swedish healthcare context where ERAS programs for JRS nowadays are considered common ground.

With no preselection of patients, all PKR cases by one high-volume surgeon will chronologically be scheduled as the first morning case during one consecutive year, and thereby be included in this observational study. In order for discharge, strict post-surgery criteria will have to be met. Patient characteristics will be documented and at a 3-month follow-up, percentages of discharge on day of surgery (DOS), patient satisfaction, adverse events (AEs) and readmissions for any cause will be evaluated.

The thesis is that the same-day surgery will be both feasible and safe as the general conception in the medical team prior to this study has been that the PKR patients, everything else equal, above all are more confident with their knee post-surgery than those that have had a TKR procedure done. As the protocol has become standard practice, the aim of this study will be to observe, document, and analyse the overall positive effects we believe this new routine has achieved.

Background and rationale

It is fair to say that a general shortening of LOS, including JRS, started off in the US, where the healthcare structure and financial environment differs substantially from e.g. Europe. Henrik Kehlet, a Danish orthopedic surgeon, is considered the first in trying to target all aspects of the perioperative care with the aim of reducing cost and complications,[11, 12] including the use of modern anesthetic techniques and multimodal analgesia with less morphine utilization.[6, 14, 20] These programs come in many names, such as Fast-track, Rapid Recovery or Enhanced Recovery after Surgery (ERAS) as often referred to in Europe where they have been widely spread, not least through JRS register channels.[2, 9, 13, 15]

The PKR procedure may be considered a candidate for same-day surgery, especially as the procedure seems to have a lower risk of short-term complications than does TKR.[1] A recent US database study comprising 169,406 patients who had had a JRS done reported no difference in readmission when comparing the inpatient and the outpatient group, even after adjusted for comorbidity.[7] Data on PKR exclusively is hard to find, but one large retrospective study by Bovonratwet et al[4] compared 568 outpatient and 5312 inpatient PKR cases between 2005 and 2015 which reported no difference in 30-day readmission rate and concluded that same-day surgery can be considered in carefully selected patients. One recent randomized controlled trial (RCT) of 40 patients have shown similar results,[10] as have observational studies.[3, 5, 8, 19]

One observational study from the US by Berger et al showed already ten years ago that 94% of 25 consecutive PKR cases were discharged on DOS with no AE/readmission during a 3-month follow-up.[3] The same group have later reported similar results in 105 unselected cases, including 14 patients classified as ASA 3.[8] Two recent PKR studies, each with their unique pre-selected inclusion criteria, one from the United Kingdom NHS[5] that included 72 patients and one from France[19] that included 50 patients, reported 85% and 94% discharge

on DOS respectively. One RCT from the Netherlands compared same-day surgery to a more traditional fast-track protocol in 40 pre-selected PKR cases and reported 90% discharge on DOS in the same-day surgery sub-group.[10]

Although it is hard to coin one definition of a fast-tack/rapid recovery/ERAS program, perhaps it is not even to strive for as every practice has its own unique settings. Though, the essence is to find a common thread in the perioperative regime in order for optimizing patient safety and comfort, with unit cost-efficiency at the same time.

As of today, still very little is written about same-day surgery procedures in the literature. With the primary objective to examine the evidence regarding the safety and feasibility of performing THR, TKR, or PKR in an outpatient setting a systematic review was recently conducted by Pollac et al; out of 805 studies only 17 passed the inclusion criteria, neither of which were randomized controlled trials, only four were controlled but lacking patient characteristics and therefore, needless to say, unadjusted.[17]

Therefore, the intention is to conduct an observational study on efficacy and safety in patients receiving a PKR surgery following a same-day surgery protocol in a Swedish healthcare context, where ERAS programs for JRS nowadays are considered common ground.

Objectives

Primary aims

The primary aims of this 3-month follow-up study to assess safety outcome measures for a same-day PKR surgery protocol, including AE and readmission for any cause.

Secondary aims

Secondary aims of this study will be to assess efficacy measures such as who and how many will be discharged on DOS, and what burden will instead be allocated to the out-patient department.

Patients and methods

Study design

Cohort study – Level of evidence, 2

This observational study will be conducted at Trelleborg hospital, a public hospital in Region Skåne, a county council in the south of Sweden, performing around 1.500 JRSs each year. The efficacy and safety aspects of the same-day surgery protocol to be assessed is in fact only a slight modification of an existing ERAS protocol that has been used for years at the department.

To best evaluate these measures, the study is pragmatically design as no patient-selection will be done prior to surgery. I.e., all patients, regardless of any medical history, considered for a PKR procedure will also be informed prior to surgery by the multidisciplinary team (nurse, surgeon, anaesthesiologist, and physiotherapist) that if the surgery is to be scheduled in the morning, he/she will then follow a same-day surgery protocol and will likely be discharged to home later the same day.

The actual "recruitment" will be done in the following manner: All of one high-volume surgeon´s first morning cases during one consecutive year will be dedicated to a PKR patient, and thereby also included in this cohort study. The slots will filled continuously as the patients are put up for surgery. When all slots have been occupied within one week any additional PKR cases will be assigned to surgery later in the day in the same week, as will all the TKRs and the THRs this year, and consequently not taking part in the study. (It must be emphasized that, for the fairness of all patients, the operation scheduling will be put

together on a weekly bases, in a strict chronological order, by a team of nurses and without any insight of the surgeon in question.)

A slight modification to an existing ERAS protocol will be used (see below section *"The same-day surgical protocol (in short)"*), in which the patients will be discharged to home on DOS if certain postoperative criteria are met. These are categorized into four dimensions; vital parameters, urinary function, bleeding and mobilization: The vital parameters will be measured and scored by the National Early Warning Score (NEWS)[16], where the threshold for discharge will be set to zero (NEWS 0). The urinary function algorithm for discharge on DOS is, in short, if no need for catheterization less than 200 ml of residual volume after spontaneous void. A compression stocking will be removed six hours post-surgery and if no ongoing bleeding discharge can be considered. Last but not least, a physiotherapist will evaluate whether basic activity of daily living (ADL) can be performed in a safely manner. The surgeon will phone the patient the day after surgery for an early check. At 3-month follow-up an evaluation of efficacy, AE and readmission will be done.

Patients

Inclusion criteria

- With no preselection of patients, all of one surgeon's UKA morning cases during one consecutive year

Exclusion criteria

- Patients who will have a UKA procedure done by any other surgeon
- Patients who will have a simultaneous bilateral UKA procedure
- Patients not scheduled as the first morning case*

**The scheduling procedure will be done in a strictly chronological manner on a weekly bases without any involvement of the surgeon/author.*

Withdrawal criteria

Not applicable

Perioperative conditions

The operating room and medical regimes

The operations will be performed using a clean-air enclosure with vertical air-flow. Three doses of intravenous antibiotic will be given each patient within the first 24 hours of surgery starting preoperatively (a total of 6 g isoxazolylpenicillin or if allergic towards penicillin 1.2 g clindamycin), as well as corticosteroids (8 mg of betamethasone) and antifibrinolytics (10 mg/kg of tranexamic acid). 300 ml of local infiltration analgesia (LIA) will be administered (300 mg of ropivacaine, 30 mg of ketorolac and 0.5 mg of adrenaline)[18]. Each patient will from day 1 be given subcutaneous injections of 40 mg low-molecular-weight heparin (enoxaparinnatrium) as thromboembolic prophylaxis.

Implant design and surgical technique

The implant design to be used in this study will be the LINK® Sled prosthesis, Link. The procedures will be done using a mini incision, i.e. not everting the patella.

The same-day surgical protocol (*in short*)

Planning stage

At the routine medical check 2 weeks prior to surgery the medical team will be notified of which patients will be scheduled for morning surgery and the patients will be given both oral and written information about the same-day surgery routine.

Day of surgery

The perioperative protocol is aimed at optimizing for rapid mobilization by avoiding early postoperative numbness (general anesthesia), urinary retention (no catheter), bleeding (antifibrinolytics), stress (corticosteroids) and discomfort (local infiltration anesthesia).

The dimensions and their respective criteria for discharge are the following:

- Vital parameters; measured and scored by the National Early Warning Score (NEWS), with the threshold set to NEWS 0
- Urinary function; less than 200 ml of residual volume after spontaneous void will be required
- Bleeding; no wound leakage will be allowed
- Mobilization; a physiotherapist will make sure the patient is able to perform ADL in a safe manner before discharged can be considered

Day after surgery

The surgeon will phone the patient to answer any questions that may have arisen since the surgery and to evaluate the following variables:

- If still positive to have been discharged on DOS, i.e. would have chosen it again and could recommend it to others?
- Degree of wound leakage?
- Degree of pain?

3-month follow-up

Patient characteristics such as age, sex, BMI, ASA and Charnley class will be used in this study. Apart from whether or not the patient did discharge on DOS and his/her experience from the routine (evaluated by a phone the day after surgery), at 3-month post-surgery the following parameters will also be evaluated:

- Any additional appointments to the outpatient department
- Any complications at the 3 month doctor's appointment
- 30-day adverse events and/or readmission for any cause
- 90-day adverse events and/or readmission for any cause

Patient information

This is an observational study with the aim to document a routine practice. I.e., the patients will be well informed of the same-day surgery routine, but not asked to participate. The reason is, without the risk of bias, to be able to thoroughly evaluate and perhaps improve the routine in any way.

This study gives us the opportunity to observe and analyse an authentic scenario, as it would e.g. be incorrect only to include the most positive individuals.

Data analysis

Statistics

Since the same-day surgery routine has become standard practice and that this observational study is conducted to demonstrate just that, no comparative analyses are planned. Only if unexpected failures will be observed, retrospectively such a comparison will be conducted. (If so, a new retrospective case control study would then be needed.)

Descriptive data will be presented as unadjusted means with standard deviations, medians with range, or as proportions (%).

Ethical considerations

Ethics approval

Approval from the Swedish Ethical Review Authority will be applied for.

Conventional X-ray

No additional radiological examinations will be performed to the ordinary routine.

Administrative procedures

Data management

The clinical investigator will be responsible for the compilation and statistical treatment of data.

Reports and publication of results

It is anticipated that the results of this study will be published in an international journal of orthopaedics. In this context the results of the study are owned solely by the clinical investigator, who decides on the final form of the report.

Timeline

All the data collection, the statistical analysis and writing the manuscript is anticipated to be done within one year from study start (2020).

Study centres

All the operations will be performed at Trelleborg hospital, a public hospital within the general health care system of Region Skåne.

Surgeons

One single high-volume surgeon will be performing all the operations.

Conflicts of interest

None

Disclosures

The investigator (surgeon) has no relevant financial or nonfinancial relationships related to the study.

References

1. Beard DJ, Davies LJ, Cook JA, MacLennan G, Price A, Kent S, et al. The clinical and cost-effectiveness of total versus partial knee replacement in patients with medial compartment osteoarthritis (TOPKAT): 5-year outcomes of a randomised controlled trial. *Lancet*. 2019 Aug 31;394(10200):746-56.
2. Berg U, BuLow E, Sundberg M, Rolfson O. No increase in readmissions or adverse events after implementation of fast-track program in total hip and knee replacement at 8 Swedish hospitals: An observational before-and-after study of 14,148 total joint replacements 2011-2015. *Acta Orthop*. 2018 Oct;89(5):522-7.
3. Berger RA, Kusuma SK, Sanders SA, Thill ES, Sporer SM. The feasibility and perioperative complications of outpatient knee arthroplasty. *Clin Orthop Relat Res*. 2009 Jun;467(6):1443-9.
4. Bovonratwet P, Ondeck NT, Tyagi V, Nelson SJ, Rubin LE, Grauer JN. Outpatient and Inpatient Unicompartmental Knee Arthroplasty Procedures Have Similar Short-Term Complication Profiles. *J Arthroplasty*. 2017 Oct;32(10):2935-40.
5. Bradley B, Middleton S, Davis N, Williams M, Stocker M, Hockings M, et al. Discharge on the day of surgery following unicompartmental knee arthroplasty within the United Kingdom NHS. *Bone Joint J*. 2017 Jun;99-b(6):788-92.
6. Burn E, Edwards CJ, Murray DW, Silman A, Cooper C, Arden NK, et al. Trends and determinants of length of stay and hospital reimbursement following knee and hip replacement: evidence from linked primary care and NHS hospital records from 1997 to 2014. *BMJ open*. 2018 Jan 27;8(1):e019146.
7. Courtney PM, Boniello AJ, Berger RA. Complications Following Outpatient Total Joint Arthroplasty: An Analysis of a National Database. *J Arthroplasty*. 2017 May;32(5):1426-30.
8. Cross MB, Berger R. Feasibility and safety of performing outpatient unicompartmental knee arthroplasty. *Int Orthop*. 2014 Feb;38(2):443-7.
9. Glassou EN, Pedersen AB, Hansen TB. Risk of re-admission, reoperation, and mortality within 90 days of total hip and knee arthroplasty in fast-track departments in Denmark from 2005 to 2011. *Acta Orthop*. 2014 Sep;85(5):493-500.
10. Hoorntje A, Koenraadt KLM, Boeve MG, van Geenen RCI. Outpatient unicompartmental knee arthroplasty: who is afraid of outpatient surgery? *Knee Surg Sports Traumatol Arthrosc*. 2017 Mar;25(3):759-66.
11. Kehlet H. Fast-track surgery-an update on physiological care principles to enhance recovery. *Langenbecks Arch Surg*. 2011 Jun;396(5):585-90.
12. Kehlet H. Multimodal approach to control postoperative pathophysiology and rehabilitation. *Br J Anaesth*. 1997 May;78(5):606-17.
13. Khan SK, Malviya A, Muller SD, Carluke I, Partington PF, Emmerson KP, et al. Reduced short-term complications and mortality following Enhanced Recovery primary hip and knee arthroplasty: results from 6,000 consecutive procedures. *Acta Orthop*. 2014 Feb;85(1):26-31.
14. Kim S, Losina E, Solomon DH, Wright J, Katz JN. Effectiveness of clinical pathways for total knee and total hip arthroplasty: literature review. *J Arthroplasty*. 2003 Jan;18(1):69-74.
15. Pamilo KJ, Torkki P, Peltola M, Pesola M, Remes V, Paloneva J. Reduced length of uninterrupted institutional stay after implementing a fast-track protocol for primary total hip replacement. *Acta Orthop*. 2018 Feb;89(1):10-6.

16. Physicians RCo. National Early Warning Score (NEWS) - Standardising the Assessment of Acute-Illness Severity in the NHS. *London, England: Royal College of Physicians*. 2012.
17. Pollock M, Somerville L, Firth A, Lanting B. Outpatient Total Hip Arthroplasty, Total Knee Arthroplasty, and Unicompartmental Knee Arthroplasty: A Systematic Review of the Literature. *JBJS reviews*. 2016 Dec 27;4(12).
18. Rostlund T, Kehlet H. High-dose local infiltration analgesia after hip and knee replacement--what is it, why does it work, and what are the future challenges? *Acta Orthop*. 2007 Apr;78(2):159-61.
19. Ruiz N, Buisson X, Filippi G, Roulet M, Robert H. Ambulatory unicompartmental knee arthroplasty: Short outcome of 50 first cases. *Orthopaedics & traumatology, surgery & research : OTSR*. 2018 Nov;104(7):961-6.
20. Stambough JB, Nunley RM, Curry MC, Steger-May K, Clohisy JC. Rapid recovery protocols for primary total hip arthroplasty can safely reduce length of stay without increasing readmissions. *J Arthroplasty*. 2015 Apr;30(4):521-6.
