# Supplementary material for: On the generalizability of same-day partial knee replacement surgery—A non-selective interventional study evaluating efficacy, patient satisfaction, and safety in a public hospital setting
Source: PLoS One. 2021 Dec 7;16(12):e0260816. doi: 10.1371/journal.pone.0260816 (PMC8651131; doi:10.1371/journal.pone.0260816)
Supplement: S2 Appendix — (DOCX) [file pone.0260816.s002.docx]

**S2. Supplementary information**

**On the Generalizability of Same-day Partial Knee Replacement Surgery – A non-selective Interventional Study evaluating Efficacy, Patient Satisfaction, and Safety in a public Hospital Setting**

**S2a. Response categories for anchor type questions concerning satisfaction and wound leakage.**

The patients were asked about their feelings concerning the same-day surgery experience. They were told to give a “yes” or “no” answer to the following statements: “A positive experience; would do it again; can recommend to others”.

(This was analyzed as a binary outcome, yes/no.)

The patients were asked to describe the bandage regarding saturation and were given the following five alternatives:

| Grade 1 | No or small stain |
| --- | --- |
| Grade 2 | <50 % wet |
| Grade 3 | >50 %, dry |
| Grade 4 | >50 %, wet |
| Grade 5 | 100 %, wet |

(This was analyzed as an ordinal outcome.)

**S2b. Response to a Numerical Rating Scale (NRS) for pain.**

The patients were asked to choose a number between 0 and 100 that best fitted their pain intensity

(This was analyzed as a numerical outcome.)
