# Supplementary material for: On the generalizability of same-day partial knee replacement surgery—A non-selective interventional study evaluating efficacy, patient satisfaction, and safety in a public hospital setting
Source: PLoS One. 2021 Dec 7;16(12):e0260816. doi: 10.1371/journal.pone.0260816 (PMC8651131; doi:10.1371/journal.pone.0260816)
Supplement: S3 Appendix — (DOCX) [file pone.0260816.s003.docx]

INTERVJUFRÅGOR

Ca 24 timmar efter operationen kommer FP ringas upp i hens eget hem. Förväntad effekt av given lokalbedövning under operationen kommer sedan flera timmar ha varit försvunnen. Naturligt förekommande ADL, så som att eventuell behöva stiga upp på natten, morgonrutiner, frukost och lunch kommer vid samtalet vara i färskt minne (och förväntas inte bli ”värre”). Förutom att undersöka den spontana fysiska och psykiska reaktionen i direkt anslutning till det dagkirurgiska protesingreppet, så kommer även graden av genomblödning i förbandet och graden av smärta att evalueras – faktorer som riskerar att lyfta resurser från sluten- till öppenvård, i form av extra mottagningsbesök.

### Fråga 1:

*”Spontant, känner du dig trygg med att ha åkt hem redan igår och att ha sovit hemma första natten, skulle du göra det igen, och rekommenderar du det till andra?”*

Svar: Ja/Nej

### Fråga 2:

*Har förbandet blött igenom, beskriv blödningen? (Om tveksamhet i svaret kommer mer specifika frågor ställas för att närmast beskriva eventuell blödning enligt graderingen nedan.)*

Svar:

| Grad 1 | Ingen eller punktblödning |
| --- | --- |
| Grad 2 | <50 % mättat |
| Grad 3 | >50 % mättat, torrt |
| Grad 4 | >50 % mättat, blött |
| Grad 5 | 100 % mättat |

### Fråga 3:

*På en skala noll till hundra, hur ont har du nu? (Om diskussion uppkommer kring innan eller efter att hen har tagit sitt analgetikum, så kommer symptomen efter att ha tagit det efterfrågas.)*

Svar: Symptomen kommer att presenteras i form av NRS skala (0–100)
